# SwissDock 2024: major enhancements for small-molecule docking with Attracting Cavities and AutoDock Vina

**DOI:** 10.1093/nar/gkae300

**Published:** 2024-04-30

**Authors:** Marine Bugnon, Ute F Röhrig, Mathilde Goullieux, Marta A S Perez, Antoine Daina, Olivier Michielin, Vincent Zoete

**Affiliations:** Molecular Modeling Group, SIB Swiss Institute of Bioinformatics, CH-1015 Lausanne, Switzerland; Molecular Modeling Group, SIB Swiss Institute of Bioinformatics, CH-1015 Lausanne, Switzerland; Molecular Modeling Group, SIB Swiss Institute of Bioinformatics, CH-1015 Lausanne, Switzerland; Molecular Modeling Group, SIB Swiss Institute of Bioinformatics, CH-1015 Lausanne, Switzerland; Molecular Modeling Group, SIB Swiss Institute of Bioinformatics, CH-1015 Lausanne, Switzerland; Molecular Modeling Group, SIB Swiss Institute of Bioinformatics, CH-1015 Lausanne, Switzerland; Department of Oncology, Geneva University Hospital (HUG), CH-1205 Geneva, Switzerland; Molecular Modeling Group, SIB Swiss Institute of Bioinformatics, CH-1015 Lausanne, Switzerland; Department of Oncology UNIL-CHUV, Ludwig Institute for Cancer Research, Lausanne Branch, University of Lausanne, CH-1015 Lausanne, Switzerland

## Abstract

Drug discovery aims to identify potential therapeutic compounds capable of modulating the activity of specific biological targets. Molecular docking can efficiently support this process by predicting binding interactions between small molecules and macromolecular targets and potentially accelerating screening campaigns. SwissDock is a computational tool released in 2011 as part of the SwissDrugDesign project, providing a free web-based service for small-molecule docking after automatized preparation of ligands and targets. Here, we present the latest version of SwissDock, in which EADock DSS has been replaced by two state-of-the-art docking programs, i.e. Attracting Cavities and AutoDock Vina. AutoDock Vina provides faster docking predictions, while Attracting Cavities offers more accurate results. Ligands can be imported in various ways, including as files, SMILES notation or molecular sketches. Targets can be imported as PDB files or identified by their PDB ID. In addition, advanced search options are available both for ligands and targets, giving users automatized access to widely-used databases. The web interface has been completely redesigned for interactive submission and analysis of docking results. Moreover, we developed a user-friendly command-line access which, in addition to all options of the web site, also enables covalent ligand docking with Attracting Cavities. The new version of SwissDock is freely available at https://www.swissdock.ch/.

## Graphical Abstract

**Graphical Abstract:**
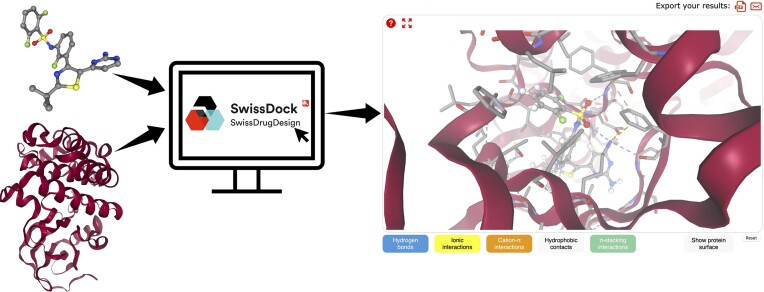

## Introduction

Identifying and optimizing potential therapeutic molecules in drug discovery is a multifaceted undertaking that requires innovative computational approaches. Molecular docking is one of the most widely used structure-based drug design methods (1–3). It involves the computational prediction of the preferred position, orientation, and conformation (i.e. poses) of a real or putative ligand within the binding site of a target biomacromolecule. The latter are mainly proteins, but other types of macromolecules, such as nucleic acids or polysaccharides can also be encountered. Docking consists in generating multiple poses of the small molecule at the surface of the target and determining the most favorable pose in terms of ligand-target interaction energy, conformational energy and possibly also desolvation energy. Ideally, but not necessarily, the docking software can estimate the small molecule binding free energy for the target to facilitate the identification of potential ligands. This predictive modeling approach speeds up the drug discovery process and reduces experimental costs. Molecular docking is based on the fundamental principles of molecular recognition, which is essentially governed by non-covalent interactions. However, covalent inhibitors are attracting increasing interest due to their ability to form stable interactions with their target, leading to potent and enduring inhibition (4). The scoring functions used in molecular docking simulations strive to accurately represent these interactions, enabling a vast chemical space to be explored and ligands with optimal binding affinity to be identified. Many molecular docking algorithms exist, some well-known examples include our in-house algorithms EADock Dihedral Space Sampling (DSS) (5) and Attracting Cavities (6,7), or AutoDock and Autodock Vina (8–11). Several web tools give access to docking algorithms, such as DockingServer (https://www.dockingserver.com/web/), Webina (12), DOCK Blaster (13), ParDOCK (14), SeamDock (15) or ProteinsPlus (16).

SwissDock is a computational tool created in 2011 as part of the SwissDrugDesign project (17,18). Its first version, freely accessible without login, is based on the EADock DSS docking engine (5). SwissDock automatically prepares the ligand and the target for docking. Calculations are performed on a server and require no computational power on the part of the client. Docking results are displayed on an interactive web page, giving users an overview of ligand poses on the surface of the target in 3D. Since its creation, SwissDock has been used by 530 000 users from about 200 countries, who performed >710 000 dockings. The use of SwissDock has grown steadily, particularly during the SARS-CoV-2 crisis (19,20), and has generated around 1200 citations by January 2024, according to Clarivate.

Here, we present SwissDock 2024, which constitutes a major update of our web tool. Docking calculations can now be done using either our in-house algorithm Attracting Cavities 2.0 (7) or AutoDock Vina 1.2.5 (10). AutoDock Vina yields good accuracy docking predictions with fast execution times, while Attracting Cavities provides higher confidence predictions with longer calculation times. The preparation of the ligand and target is automatized for both docking algorithms without the need of any prior manipulation by users. Our web interface has been revamped as for other SwissDrugDesign tools, such as SwissParam (21), SwissSimilarity (22) and SwissBioisostere (23). The SwissDock 2024 web interface is easy to use and interactive. Ligands can be imported in several formats, such as SMILES notation and Tripos Mol2 files, or drawn and visualized in 2D in a molecular sketcher. Targets can be imported as a Protein Data Bank (PDB) file or identified through their PDB ID and visualized in 3D. In addition, input data can be retrieved from several databases, such as ChEBI (24) and the chemical component dictionary from the worldwide Protein Data Bank (25,26) for ligands, as well as PDBe (27), the AlphaFold protein structure database (28) or SWISS-MODEL (29) for targets, providing easy access to a wealth of high-quality structural data. Users can monitor the progress of their docking jobs and possibly cancel them. Results are automatically displayed on an interactive page, where ligand poses are visualized in 3D, and several types of ligand-target interactions displayed on demand. Ligands can also be submitted with a simple click to other tools of the SwissDrugDesign suite, like SwissParam (21), SwissTargetPrediction (30), SwissADME (31), SwissSimilarity (22) and SwissBioisostere (23). Finally, users can submit, monitor and retrieve docking calculations through a new, easy-to-use command-line access. All the options of the graphical interface are included in the command-line, which includes two additional capacities (i) the docking of covalent ligands using the *switch* method of Attracting Cavities (32) and (ii) the inclusion of crystallographic water molecules as integral part of the target structure. SwissDock 2024 is accessible at https://www.swissdock.ch/, while the previous version will remain available for at least six months at http://old.swissdock.ch/. SwissDock 2024 is freely accessible without login, and results are provided under CC BY 4.0 license.

## Materials and methods

### Docking algorithms

The first version of SwissDock performed molecular docking using the EADock DSS algorithm (5,17). SwissDock 2024 is based on the latest version of Attracting Cavities (AC) (6,7,32) and also offers the user the choice to perform faster molecular docking with AutoDock Vina (9,10).

#### Attracting cavities 2.0 *(AC)*

The first step of the AC docking algorithm consists in positioning attracting, placement, and electrostatic cloud points in the cavity of the macromolecule. A cavity prioritization parameter determines the level of concavity of the cavities considered for docking. A threshold of 70 focuses the docking on deep binding cavities, while a value of 50 includes shallower clefts. After positioning the attracting, placement, and electrostatic points in the macromolecule cavities, the target is removed, leaving only the cloud points. To be docked in this ‘mold’, the ligand is successively positioned on each placement point, then rotated around the x, y and z axes according to the sampling exhaustivity parameter that determines the rotational angle step. The smaller the rotational angle step, the greater the number of poses considered. Moreover, AC 2.0 allows for random modification of the initial ligand orientation and box center to limit the dependence on the starting conditions. A geometry optimization of the ligand in the ‘mold’ is performed before removing the cloud points and reintroducing the target macromolecule. This is followed by geometry minimizations of the ligand in the target, first with a softcore potential and then with the standard potential. AC 2.0 has two different scoring functions: (i) the AC docking score, which consists of the CHARMM force field energy plus the FACTS solvation energy terms (33–35), and (ii) the SwissParam score, which provides an estimate of the binding free energy as a weighted sum of the polar and nonpolar terms (36). Ligand poses are ranked according to their AC docking score, while the SwissParam score is provided to enable the comparison of multiple putative ligands for the same target. For covalent ligand-protein docking, the sampling utilizes the non-covalent pre-reactive ligand topology, whereas pose refinement and scoring are carried out using the post-reactive topology, encompassing the covalent bond formed with the protein (32).

#### AutoDock Vina

We implemented AutoDock Vina in SwissDock 2024 using the Vina Python library (version 1.2.5) (10). The Python interface allows to create a Vina sampling engine and to have access to the scoring functions of Vina, AutoDock4 (8), and Vinardo (11). For simplicity, SwissDock 2024 currently only uses the Vina scoring function, which comprises a term to account for van der Waals interactions, an undirected hydrogen-bond component, a hydrophobic factor and a penalty for conformational entropy. Vina computes interactions between molecules through trilinear interpolations of precalculated grid maps on the target structure inside a search box whose center and size are provided by the user. Additionally, it uses the target structure for postprocessing minimization of the docked poses. In the SwissDock output, ligand poses are ranked according to their Vina score.

Attracting Cavities 2.0 and AutoDock Vina have undergone comprehensive benchmarking to evaluate their performance (7,10,32). These studies offer insights into the accuracy and efficiency of both algorithms in predicting ligand–receptor binding.

### Input file preparation

As AC requires CHARMM-formatted files and Vina PDBQT files, respectively, the uploaded ligand and target structures are converted for the chosen docking algorithm to let users seamlessly input molecules in usual formats such as SMILES, Mol2 or PDB.

#### Ligand preparation

In SwissDock 2024, the ligand can be uploaded in several formats: as a Mol2 file, as a PDBQT file (Vina only), using the SMILES notation, or through the MarvinJS chemical editor. The latter accepts several other types of file formats, such as SDF or MDL Mol, and converts them to SMILES and 2D structure (ChemAxon, version 21.2.0, Budapest, Hungary, www.chemaxon.com, accessed on 25 January 2024). An advanced option allows searching for small molecules by name, PDB ID or InChI, in a combined collection—comprising the chemical component dictionary from the worldwide Protein Data Bank (25,26) and the complete ChEBI database (24)—in which compounds were already protonated at pH 7.4. For AC, ligands are prepared with the new version of SwissParam (21). Molecules, in the SMILES notation or as Mol2 files, are submitted to SwissParam through its command line interface, and the parameterization is performed using the MMFF-based approach, compatible with the CHARMM36 force field (35). For Vina, if the input ligand is not initially provided as a PDBQT file but in SMILES notation (via the sketcher or the advanced search), the latter is standardized before being converted to a Mol2 file with 3D coordinates for all atoms, using the ChemAxon JChem Microservices (version 21.3). Mol2 files, generated from the SMILES notation or directly provided by the user, are converted to PDBQT files using the *mk_prepare_ligand.py* script from the Meeko python package (version 0.5.0).

#### Target preparation

The macromolecular target can be uploaded as a PDB (AC or Vina) or as a PDBQT file (Vina only). It can also be retrieved directly from PDBe (26) by providing its PDB ID. PDB files are parsed to display information about chains and heteroatoms so that the user can select, via the SwissDock web interface, the elements to be retained for the docking. SwissDock 2024 also provides an advanced target search option in three different structural databases: PDBe (26), SWISS-MODEL (29), and the AlphaFold protein structure database (28,37). For Vina, the PDB file is converted into a PDBQT file using the *prepare_receptor* command tool from the AutoDockFR (ADFR) Suite (version 1.0) (38). For AC, PDB files are prepared using the previously developed method (17). All residues are automatically protonated at physiological pH. Protonation states and tautomers of histidine side chains are determined based on the chemical environment of the imidazole ring. Missing side chains and hydrogen atoms are added through CHARMM. Water molecules are removed automatically, and potential clashes are detected and removed. Force field parameters and topologies are taken from the CHARMM36 force field (35) for amino acids, nucleic acids, non-natural amino acids (39), post-translational modifications, and a wide range of ions. Cofactor parameters and topologies are either retrieved from CHARMM36 or a set of SwissParam (21) files precalculated for all ligands present in the Ligand Expo resource of the PDB (40). Using the command line, targets can be prepared for covalent docking by simply providing a prepared covalent ligand and the atom number of the protein atom forming the covalent bond to the ligand. The pre-reactive target topology is modified automatically by the web tool, which adds or deletes atoms and adjust bond lengths, to generate the post-reactive topology. Both the pre- and post-reactive forms of the target are prepared for CHARMM, as explained above. Moreover, the command line provides an option to retain water molecules in the target structure.

### Implementation

The frontend of the SwissDock 2024 website is implemented in HTML5, PHP 7.4.3, and JavaScript. To display ligands in the molecular sketcher, in the protonation state that will be used during docking, files are converted to SMILES notation using OpenBabel (version 2.4.1) for PDBQT files and RDKit for Mol2 files (version 2022.9.1, http://rdkit.org/). The 3D structure of the target protein and the calculated poses of the ligand are displayed on the submission and result pages, respectively, with the NGL Viewer (version 2.1.0) in JavaScript (41). Molecular interactions can be displayed for atoms selected graphically by the user. Hydrogen bonds, ionic interactions, hydrophobic contacts, as well as cation-π and aromatic interactions, can be displayed separately or together at the user's discretion. The NGL viewer is also used to define the search box graphically. Dropdown selections are implemented with sumoselect (version 3.4.9, http://hemantnegi.github.io/jquery.sumoselect). The representation of the 2D structure of the ligand on the result page is produced with the ChemAxon JChem Microservices.

Calculations are performed on our servers and, therefore, require no computing power from the user side. The backend was coded using the web.py (version 0.62) package in Python 3.8. A queuing system based on Slurm (version 19.05.5) was implemented to schedule calculations. It allows users to monitor their jobs and stop them if necessary.

## Results

SwissDock 2024 (https://www.swissdock.ch/) is a freely available and login-free docking web server. The website was tested and optimized for Google Chrome (www.google.com/chrome/, accessed on 26 January 2024), Safari (www.apple.com/safari/, accessed on 26 January 2024), and Mozilla Firefox (www.mozilla.org/firefox/, accessed on 26 January 2024). Docking results and outputs are provided under a CC-BY 4.0 International License. A ‘Tutorials’ page contains videos on how to prepare ligands and targets, how to choose parameters and check a session before starting the docking, as well as on how to interpret the results. Users can also refer to the FAQ page, which provides additional documentation on the use of SwissDock 2024. Command-line operation is described in detail in the ‘Command-line’ page. A contact page provides a form to ask for further assistance. Three ligand-target examples are provided at the top of the submission page.

### Input

To start a docking session, users are first invited to select the docking algorithm at the top of the home page. The docking algorithm can be Attracting Cavities 2.0 (AC) (7) or AutoDock Vina (Vina) (10). Once the choice has been made, a molecular docking session comprises five steps: (i) submit a ligand, (ii) submit a target, (iii) define the search space, (iv) select docking parameters and check them and (v) start the docking. They are described in further detail below.

#### Submit a ligand

**Figure 1.**
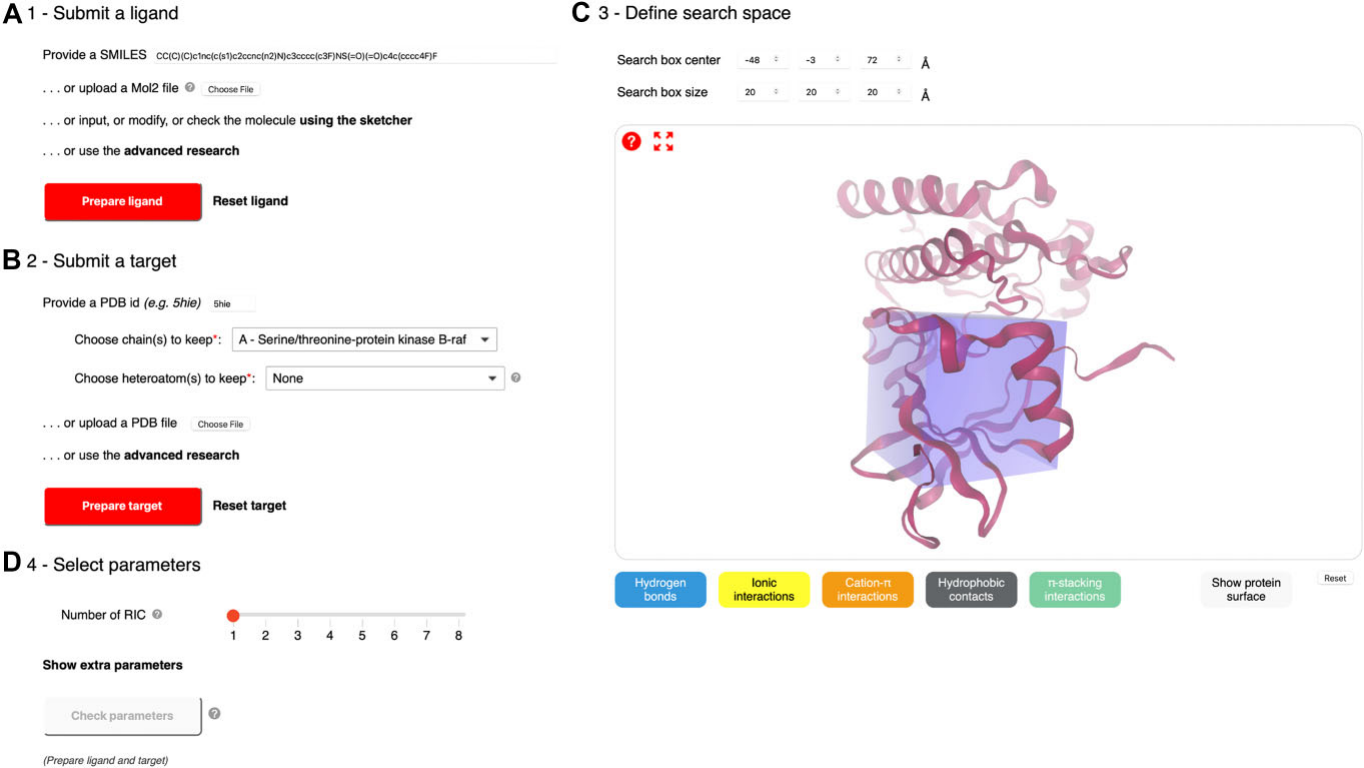
Pre-filled submission form using Attracting Cavities 2.0 and the example of dabrafenib (PDB ID P06) bound to B-Raf (PDB ID 5hie). (**A**) The submission of the ligand can be done in several ways (e.g. typing a SMILES, uploading a file, sketching, or using the advanced search). Once the ligand is selected, the ‘Prepare ligand’ button, initially colored in grey, will be activated, and colored in red. (**B**) The target can be input with a PDB ID, as a file, or through an advanced search. With a PDB ID, users can select chains and heteroatoms to keep for the docking. Once the target is selected, the ‘Prepare target’ button, initially colored in grey, will be activated, and colored in red. (**C**) The search space can be dynamically defined through the molecular viewer or using the center coordinates and size boxes. Molecular interactions, as well as the protein surface, can be shown in the 3D structure using the buttons below the viewer. (**D**) The sampling exhaustivity can be defined with a slider. Extra parameters can be displayed and defined using the link below. The ‘Check parameters’ button is activated once the ligand, and the target are prepared.

The first step in preparing a docking session is to submit a ligand (Figure 1A). This can be done in several ways. One can simply write a SMILES in the dedicated text box, upload a file in Mol2 format (and PDBQT format for Vina), use the sketcher, or use the advanced search link. The sketcher can be used to import other input formats or simply to check the molecule. Molecules provided as files appear in the text box and in the molecular sketcher for visual inspection and possible modification. Molecules selected using the advanced search are uploaded as SMILES and also displayed in the text box and the sketcher. Of note, small molecules are docked in the protonation state specified by the user. Once the ligand is selected, the ‘Prepare ligand’ button, colored in grey before the selection, becomes active and colored in red and should be clicked. If ligand preparation is successful, a check mark appears on the left of the prepare button. Otherwise, a cross and an error message appear, and the user is invited to correct the small molecule.

#### Submit a target

**Figure 2.**
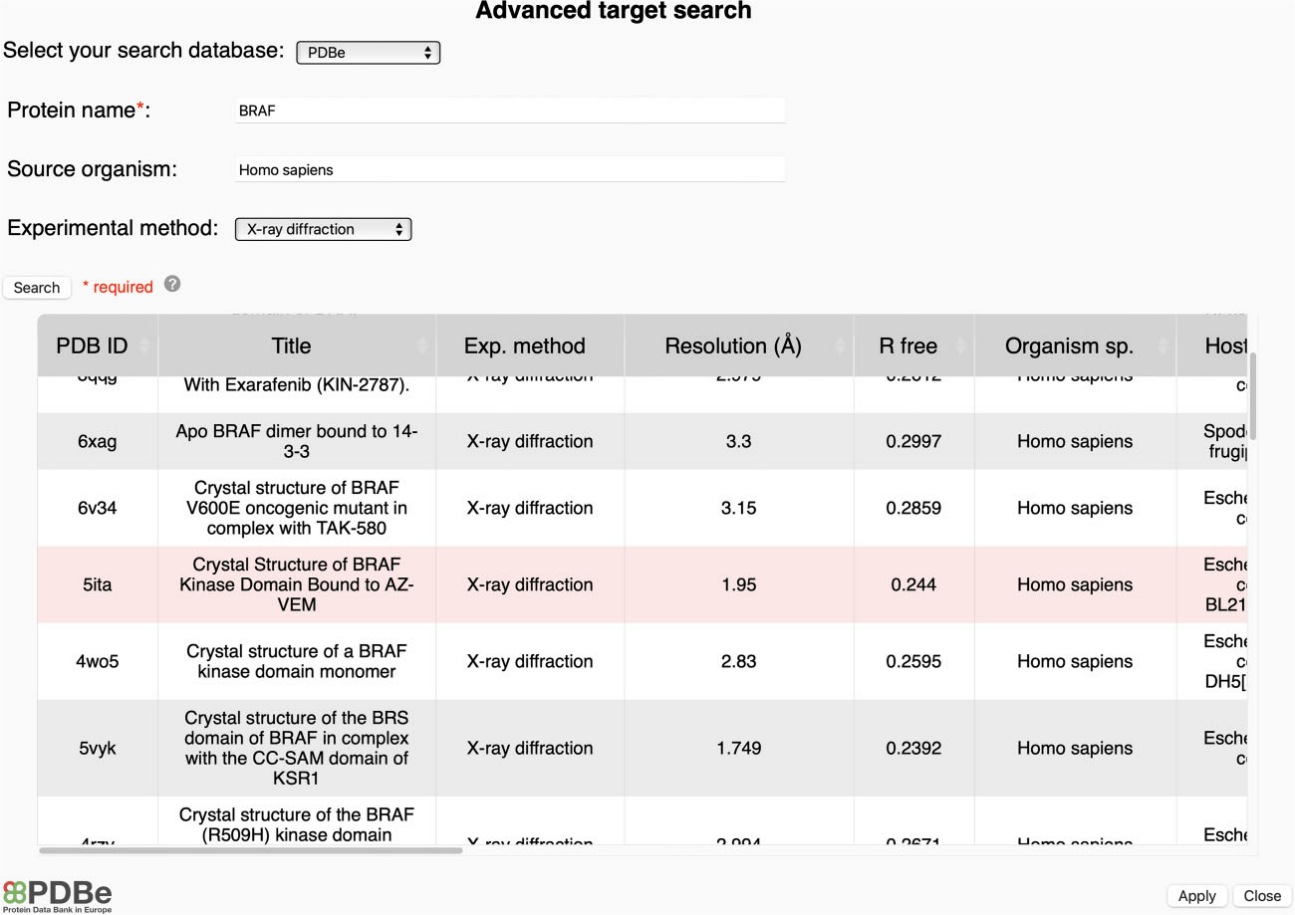
Advanced target search using the PDBe database. The database, as well as the protein name, the source organism, and the experimental method, can be chosen. In this example, entry with PDB ID 5ita is selected and can be applied in the ‘*Submit a target*’ form.

Targets can be uploaded as PDB file through the input button or by providing a PDB ID in the dedicated box (Figure 1B). If Vina was chosen as docking algorithm, users can also upload their target in the PDBQT file format. Additionally, an advanced target search is available to select and download the target as a PDB file from the chosen database (i.e. PDBe (27), SWISS-MODEL (25) or the AlphaFold protein structure database (28,37)) (Figure 2). Once the target has been uploaded, the user is invited to select the chains and heteroatoms to keep for the docking before the target preparation. Once the target is selected, the ‘Prepare target’ button, colored in grey before the selection, becomes active and colored in red. As for the ligand preparation, a check mark appears if the target preparation is successful; otherwise, a cross and an error message are displayed.

#### Define search space

The dedicated boxes can be used to define the coordinates of the center of the search space and its size in Å (Figure 1C). The center of the search space can also be defined by clicking on an atom in the molecular viewer where the target protein is displayed. Molecular interactions can be displayed by double-clicking on an atom and selecting the interaction buttons below. Possible interactions are hydrogen bonds, ionic interactions, cation-π interactions, hydrophobic contacts, and π-stacking interactions.

#### Select parameters

Users can then specify the sampling exhaustivity parameter for Vina or the number of random initial conditions (RIC) for AC using the slider (Figure 1D). Vina's sampling exhaustivity must be between 1 and 64, with a default value of 4. For AC, the number of RIC can be chosen between 1 and 8, with 1 as the default value. Moreover, extra parameters for AC can be displayed and selected using the corresponding link. The sampling exhaustivity can be chosen between *low*, *medium* (default), or *high*, which correspond to values of 180º, 90º and 60º for the rotation step, respectively (See materials and methods subsection Docking Algorithms). Available options for the cavity prioritization are *buried* (default), *medium* or *shallow*, corresponding to cavity prioritization parameter values of 70, 60 and 50, respectively. Once parameters are selected, the user verifies them by clicking the ‘Check parameters’ button. This will estimate the corresponding computational time, which must be <10 min for Vina and one hour for AC. For AC, this also checks that the number of attractive points is not equal to zero, i.e. that a cavity was found in the search space. Upon parameter validation, a check mark is displayed to the left of the button; otherwise, a cross mark and an error message are shown. The estimated calculation time is given. If estimated computation times are longer than the limits mentioned above, users are invited to adjust the different docking parameters, the search space, or the size of the target (for instance by removing a protein chain far from the cavity).

Users can specify both an email address and a docking job name, useful to get notified about job completion. Once the ligand and the target are correctly prepared and the parameters set and validated, initiating the docking process is as simple as clicking on the ‘START DOCKING’ button, which becomes active upon the completion of the required preparations described upwards.

### Output

Upon docking submission, users are automatically redirected to a waiting page displaying the docking parameters, the estimated execution time, the status of the calculation (i.e. pending, running, etc.), and a progress bar when the calculation has started. Thanks to the queuing system, users can use the ‘Stop job’ button to stop their calculations if needed. If an email address was provided, the user can close this page, and an email will be sent at the end of the calculations. Otherwise, users are invited to leave this page open until docking is complete. Once calculations are finished, the user is redirected to the results page.

**Figure 3.**
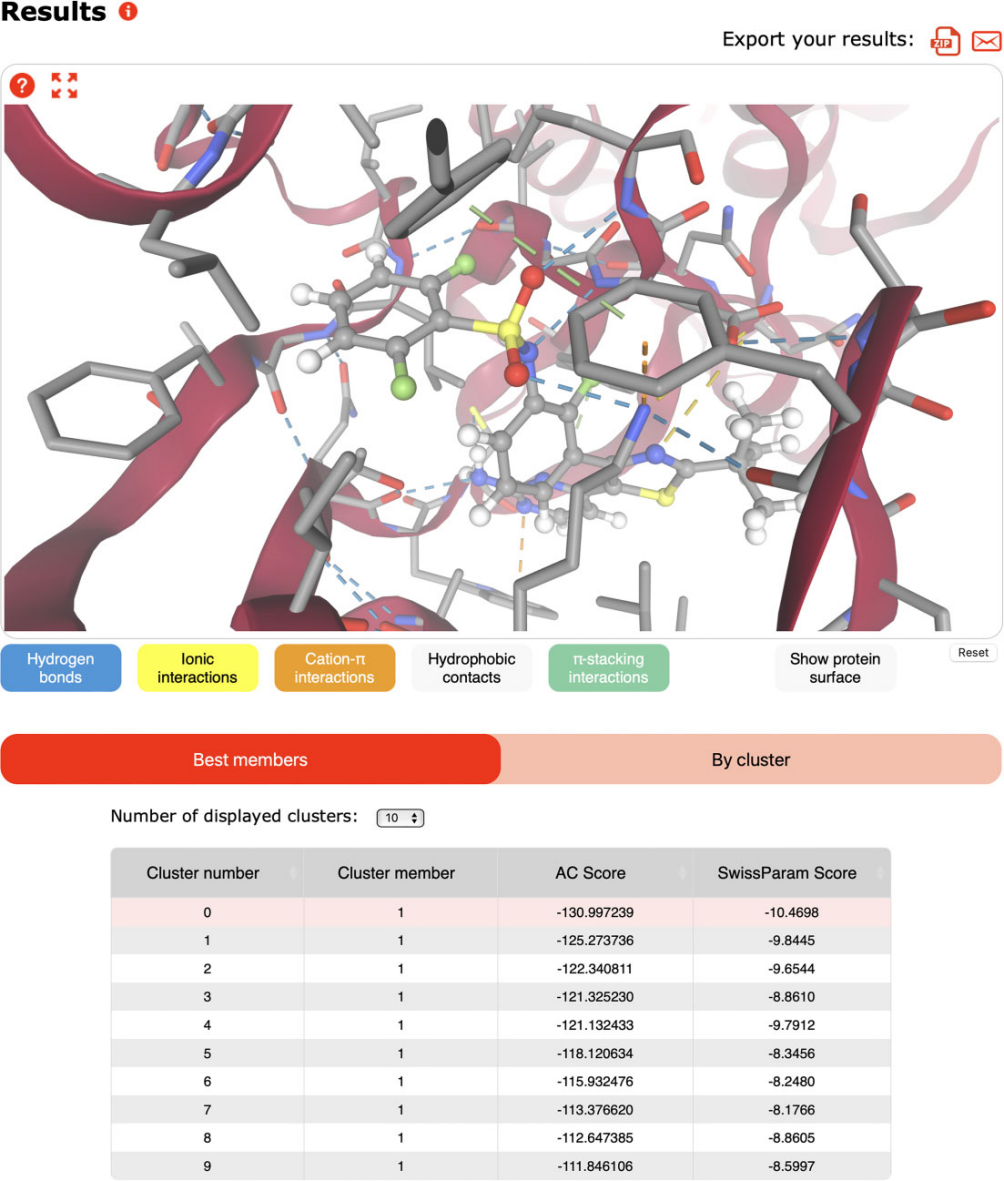
Extract from the result page of the docking of dabrafenib (PDB ID P06) to B-Raf (PDB ID 5hie) using Attracting Cavities 2.0, with a low sampling exhaustivity, a buried cavity prioritization, and a RIC of 1. The results can be downloaded as a zip file, or the URL can be copied or emailed. The target is displayed in the viewer with one or several ligand poses. The ranking of ligand poses is based on their scores, and the display can be chosen to showcase the best members of each cluster or the clusters themselves. Interactions between the target and one ligand pose are shown.

On the results page, docking parameters and a 2D structure image of the ligand are displayed. As SwissDock is now interoperable with other tools of the SwissDrugDesign suite, the ligand can be retrieved as a SMILES and/or seamlessly sent to SwissParam (21), SwissSimilarity (22), SwissTargetPrediction (30), SwissADME (31) and SwissBioisostere (23) with a single click on the corresponding interoperable icons. The results of the molecular docking are displayed below the job summary (Figure 3). Results can be retrieved as a zip file and the URL of the result page can be sent by email. The results contain the prepared ligand and target files, a parameter file summarizing the query, along with a Dock4 file for AC as well as PDB and PDBQT files for Vina containing all ligand poses (for postprocessing or advanced analysis in third-party environments, like UCSF Chimera (42) or ChimeraX (43)). The target and its heteroatoms are displayed in the NGL viewer, followed by a table containing the ligand poses ranked according to the docking score. If the docking was done using AC, users can choose between a table containing the best pose of each cluster and a table with all poses within a cluster. For AC, users can choose to rank the poses either by the docking score or by the SwissParam score by clicking on the corresponding entry in the table header. For Vina, only one table containing the poses ranked by their docking score is shown. Ligand poses can be displayed in the viewer by clicking on the corresponding row in the table. If one pose is selected, its 3D structure will appear in the viewer, and a zoom will show the molecular interactions between the pose and the target protein. Results are stored on the server for at least a week before being deleted.

### Command-line access

SwissDock is now fully accessible via an easy-to-use command-line access. The steps to perform molecular docking are the same as for the web interface: prepare the ligand and the target, set the parameters, check the session, and start the docking. Ligands can be uploaded as Mol2 files or in a SMILES format. It is possible to directly upload a PDBQT file for Vina and a zip file containing ligand parameters and topology files for AC. The target can be uploaded in PDB format for both algorithms, in PDBQT format for Vina, or as a zip file containing a coordinate file and a protein structure file in CHARMM format for AC. Crystallographic water molecules present in the target file can be kept during the preparation. Checks done on the session are the same as on the web interface, to ensure that target and ligands are ready to be docked. However, unlike the web interface, the parameters can be freely chosen if the estimated calculation time does not exceed 1 hour for AC or 10 min for Vina. During the calculations, users can check their job status by a single command. A command to download a results zip file is given to users at the end of the calculation. Users can refer to the ‘Command-line’ page on the SwissDock website for all detailed information.

If a covalent docking with AC is performed, the ligand must be prepared before the target. For ligand preparation, users are invited to specify the chemical reaction, the reactive protein residue type, the name of the ligand atom forming the covalent bond, as well as the provided form of the ligand (pre- or post-reactive). For target preparation, the atom number of the protein atom forming the covalent bond with the ligand is required. Further details regarding the covalent docking preparation are also given on the ‘Command-line’ webpage.

## Conclusion

We presented here the latest version of the SwissDock web tool, SwissDock 2024. The website has been revamped in line with other tools belonging to the SwissDrugDesign project (18). The web interface is now more user-friendly and interactive for providing, displaying, and analyzing inputs and results. Users can now choose between two docking algorithms, the latest version of either Attracting Cavities (7) or AutoDock Vina (10). The new command-line access additionally offers access to the covalent docking algorithm of Attracting Cavities (32). One of the major updates is that ligands and targets can be uploaded in several ways. Ligands can be provided as Mol2 or PDBQT files, in SMILES notation, or by drawing them in the sketcher. Other file formats can be used via the molecular sketcher. Targets can be uploaded in PDB or PDBQT format or identified by a PDB ID. Users can select chains and heteroatoms to keep during the docking, while water molecules can also be retained using the command-line access. In addition, ligands and targets can directly be fetched from different databases. The parameter selection is easy and visual, thanks to the ability to specify the search box in the 3D viewer. Online results display is interactive, with the possibility to show or hide multiple ligand poses within the target and to visualize their interactions in the 3D viewer.

SwissDock 2024 is the only web tool giving access to one fast and one more precise state-of-the-art docking algorithms, as well as to non-covalent and covalent small-molecule docking to biological targets including proteins, nucleic acids, and diverse cofactors, without prior preparation. Users have a wide choice of input formats for both ligands and targets, including an advanced search giving access to several widely used databases. Unlike some other online docking resources, SwissDock calculations are done on its server, so the users do not need to provide the computing power. SwissDock is tightly integrated into the SwissDrugDesign suite of online tools. With a simple click, it is possible for instance to retrieve ADME parameters of a ligand through SwissADME (31), similar small molecules through SwissSimilarity (22), and possible targets through SwissTargetPrediction (30). Inversely, these tools can provide input ligands for SwissDock.

We believe that SwissDock continues to be an essential asset for the scientific community and for drug discovery, particularly owing to its recent major upgrades. The user-friendly interface, combined with time-saving yet accurate docking algorithms, facilitates the prediction of ligand-target molecular interactions. SwissDock 2024 is freely accessible without login at https://www.swissdock.ch/ and through an easy-to-use new command-line. SwissDock belongs to the SwissDrugDesign suite owned and maintained by the Molecular Modeling Group of the SIB Swiss Institute of Bioinformatics at the University of Lausanne.

## Data Availability

SwissDock 2024 is freely accessible at https://www.swissdock.ch/. ChemAxon JChem Microservices can be accessed on the following website: https://jchem-microservices.chemaxon.com/.
